# Comment on “HIV/AIDS Awareness among VCT Clients: A Cross-Sectional Study from Delhi, India”

**DOI:** 10.1155/2015/649426

**Published:** 2015-10-27

**Authors:** Siddharudha Shivalli, Soujanya Kaup

**Affiliations:** ^1^Department of Community Medicine, Yenepoya Medical College, Yenepoya University, Mangalore, Karnataka 575018, India; ^2^Department of Ophthalmology, Yenepoya Medical College, Yenepoya University, Mangalore, Karnataka 575018, India

We read the article titled “HIV/AIDS awareness among VCT clients: a cross-sectional study from Delhi, India” by Mehra et al. [1], with curiosity. This study emphasizes the poor knowledge and prevailing misconceptions about HIV/AIDS among the clients of a VCT facility in Delhi, India. However, the following issues and concerns need to be addressed.

The authors have conducted a cross-sectional analysis at the VCT facility of a tertiary care health centre situated in Delhi, India. Two hundred consecutive clients who were ≥18 years of age and consenting for participation were included. However, the following should have been mentioned: How was the sample size calculated? When VCT monthly attendance is 1100, do 200 consecutive clients represent the VCT clients attending the study setting? Study sample adequacy and representativeness are the prerequisites to ensure the internal validity of the study findings. Hence, authors should have calculated the sample size based on the published literature or hypothesis and, instead of consecutive clients, systematic random sampling (e.g., every fifth VCT client) should have been adopted.

VCT centre [now renamed as Integrated Counseling and Testing Centre (ICTC)] is a place where a person is counseled and tested for HIV, of his own free will or as advised by a medical provider [2]. However, it is not the mandate of a VCT centre to counsel and test everyone in the general population [2]. But the authors mention that 31.5% of the VCT clients had never heard of HIV/AIDS. If they had never heard of HIV/AIDS, then how come they were in VCT for HIV/AIDS Counseling and Testing? Whom did the authors consider as a “VCT client”?

In Table 5 of the study by Mehra et al. [1], chi-square (*χ* ^2^) calculation for age and education is questionable as the sample size in “unaware” categories was very small. How many cells had an expected count less than 5? Did the authors consider Yates's correction or any other apt correction for chi-square (*χ* ^2^)? Authors should have clearly addressed this in Materials and Methods. Authors could have reduced the degree of freedom by logically clubbing the age and education subgroups.

The authors conclude that a significant proportion of Indian population is unaware of HIV/AIDS to the extent that they have not even heard of it and the prevalence of misconceptions regarding HIV transmission is also high in the Indian population. However, VCT clients do not represent the general population and extrapolating the findings of single VCT centre based study to Indian population is highly questionable [3]. In fact, authors should have stated it as a limitation.

Nonetheless, we must applaud the authors for investigating an important public health problem.
